# Clinical Efficacy of Intravitreal Ranibizumab in Early and Mid-Idiopathic Choroidal Neovascularization

**DOI:** 10.1155/2014/382702

**Published:** 2014-04-27

**Authors:** Chuanfeng Fan, Qiang Ji, Yu Wang, Xiangwen Shu, Juan Xie

**Affiliations:** Department of Ophthalmology, The Second People's Hospital of Jinan, No. 148 Jingyi Road, Jinan, Shandong 250001, China

## Abstract

*Background*. To compare visual outcomes and spectral-domain optical coherence tomography results following intravitreal ranibizumab treatment for early and mid-idiopathic choroidal neovascularization (ICNV). *Methods*. This retrospective, case-controlled study examined 44 patients with ICNV in one eye initially treated with intravitreal ranibizumab (0.5 mg). Further intravitreal treatments were administered as necessary. Patients were divided into two groups according to disease duration, that is, ≤3 months or 3–6 months (early and mid-groups), and the data were compared. *Results*. All patients completed at least 12 months of follow-up. Significant differences were observed between the groups in best-corrected visual acuity and in central macular thickness (CMT) reduction at all five follow-up visits. At the last follow-up (12 months), 19 early group eyes (79.1%) and 10 mid group eyes (50.0%) had statistically significant visual gains of >15 early treatment diabetic retinopathy study (ETDRS) letters (*χ* ^2^ = 4.130, *P* = 0.042). The mean number of injections was significantly higher  (*P* = 0.0001) in the mid group (2.53 ± 1.76) than in the early group (1.22 ± 1.01). *Conclusions*. Early intravitreal ranibizumab for ICNV can result in better visual prognoses, more obvious decreases in CMT, and fewer injections.

## 1. Introduction

Idiopathic choroidal neovascularization (ICNV) occurs in patients younger than 50 years without any other predisposition for choroidal neovascularization (CNV). The pathological basis of ICNV and age-related macular degeneration (AMD) are similar; both present as choroidal neovascularization in the macular region and cause bleeding, oozing, and fibrous scarring [1]. These pathological changes are closely related to vascular endothelial growth factor (VEGF) expression [2]. Although ICNV may have better prognosis than AMD, it occurs mainly in young people, and is therefore more devastating.

In recent years, a number of reports have shown that bevacizumab, one of the anti-VEGF monoclonal antibodies, achieved good effects in treating ICNV [3, 4]. In 2012, after ranibizumab was approved for sale in China, we used it to treat ICNV. We achieved good effects in some patients, but it had a limited effect in other patients. Therefore, we reviewed our ICNV patients and allocated them into two groups according to the length of the treatment course, the early group and the mid group, to observe the effects in the different groups and the effect of disease duration on prognosis.

## 2. Materials and Methods

From January 2012 to December 2012, 44 patients were diagnosed with ICNV in one eye with disease duration of ≤6 months. Among them were 15 men (15 eyes) and 29 women (29 eyes); the patients were aged 18–49 years with a mean age of 32.57 ± 7.13 years and a mean disease duration of 49.07 ± 17.65 days. All patients underwent a visual examination based on the early treatment diabetic retinopathy study (ETDRS) eye chart, indirect ophthalmoscopy, fundus fluorescein angiography (FFA), optical coherence tomography (OCT), and other tests depending on the duration of their disease, that is, <3 months versus 3–6 months.

**Figure 1:**
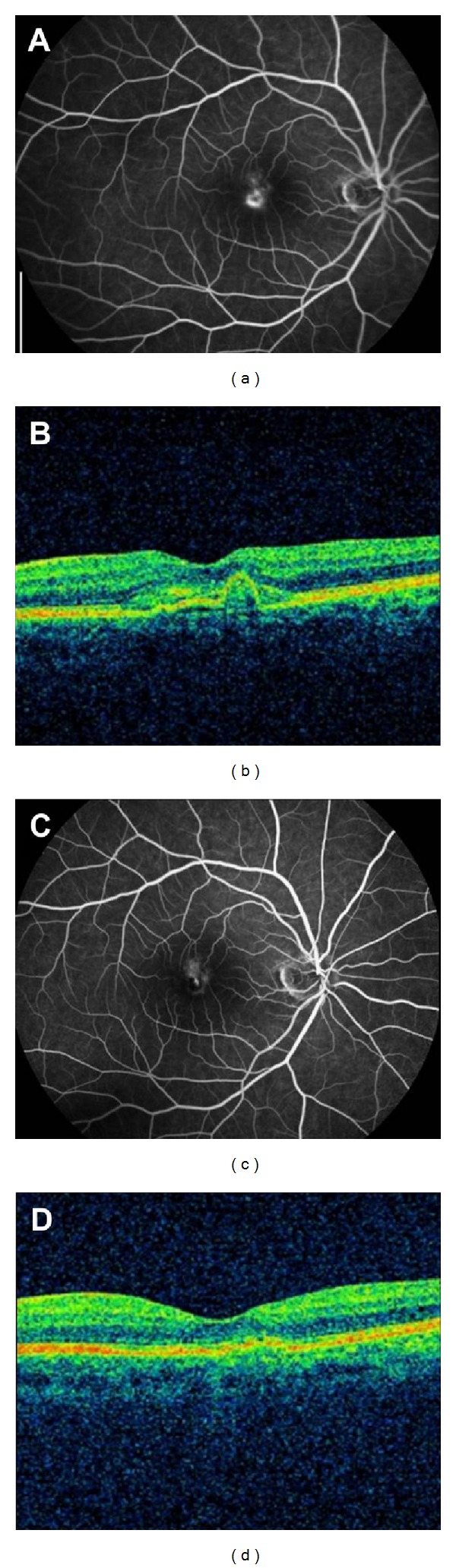
Fluorescein angiography and horizontal optic coherence tomography findings in mid and early group patients. (a) Fluorescein angiography at late stage and (b) horizontal optic coherence tomography cross-sections of the macula in the right eye of a 28-year-old woman with subretinal idiopathic choroidal neovascularization and intraretinal edema. Her best-corrected visual acuity (BCVA) was 63 letters at baseline. Twelve months later, the BCVA was 80 letters and no obvious leakage of choroidal neovascularization could be observed (c). The retinal profile was normal (d).

Inclusion criteria for ICNV in this study were (1) patients <50 years old; (2) absence of concurrent ocular diseases in the study eyes that compromised or could have compromised vision and ocular conditions; (3) no signs of pathologic myopia, including chorioretinal atrophy, posterior staphyloma, and lacquer cracks at the time of diagnosis; (4) evidence of macular CNV lesions and leakage on FFA examination; (5) a minimum follow-up period of 12 months; and (6) subfoveal or juxtafoveal CNV (Figure 1).

The exclusion criteria were (1) history of prior treatment for CNV, including laser, submacular surgery, or radiation; (2) history of a sub-Tenon capsule injection of triamcinolone acetonide, photodynamic laser treatment (PDT), or anti-VEGF injection in the 6 months before the baseline treatment of idiopathic CNV; (3) cataract surgery during follow-up; and (4) significant hepatic disease such as active hepatitis, hypersensitivity, or allergy to fluorescein (excluded because of the absence of angiographic studies) [5].

The study was approved by the hospital ethics committee. All patients were informed of the purpose, potential benefits, and possible complications of intravitreal drug therapy. When ready, subjects signed an informed consent form before each intraocular injection. All injections were performed in the operating room with a sharp 29-gauge needle. The needle was inserted into the eye through the pars plana, 3.5–4 mm from the limbus. Ranibizumab 10 mg/mL (Genentech, Inc., 0.5 mg in 0.05 mL) was intravitreally injected.

All patients returned for follow-up visits 1 day, 1 week, and 1 month after their first intravitreal injection. Patients were followed up monthly thereafter. The best-corrected vision acuity (BCVA) and intraocular pressure (IOP) (Goldman tonometer) were measured, slit lamp and indirect ophthalmoscopy were performed, and spectral-domain OCT images were collected to measure central macular retinal thickness (CMT). FFA was checked if necessary. Follow-ups were performed for 12 months.

**Figure 2:**
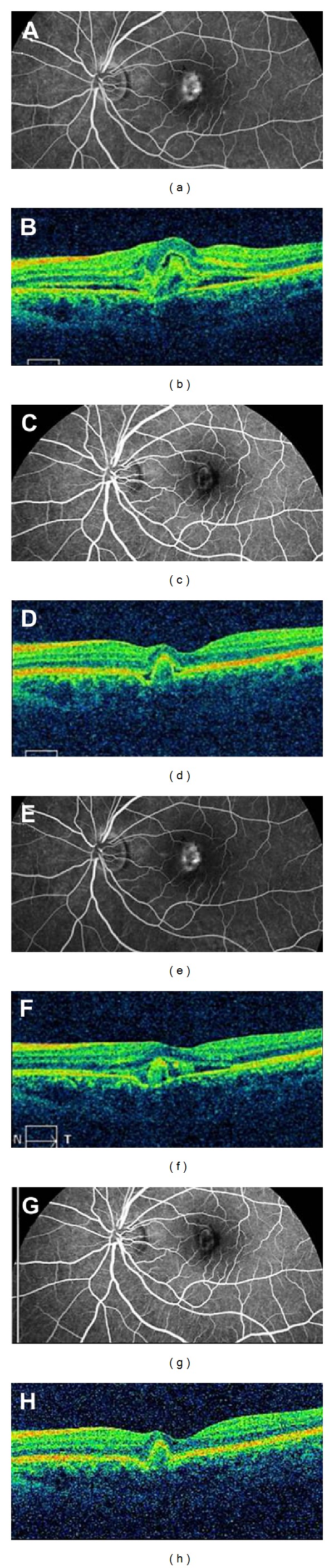
Fluorescein angiography and horizontal optic coherence tomography findings in a patient in the interim group. (a) Fluorescein angiography at late stage and (b) horizontal optic coherence tomography cross-sections of the macula from in the left eye of a 20-year-old woman with subretinal idiopathic choroidal neovascularization, subretinal fluid, and intraretinal edema. Her BCVA was 44 letters at baseline. Three months later, the BCVA was 54 letters, and subretinal fluid had reduced (c). The retinal pigment epithelium had also become smaller (d). Six months after the injection, compared to the conditions at 3 months after injection, fluorescein leakage appeared again (e), subretinal fluid increased (f), and BCVA was 49 letters. Therefore, the 2nd injection was administered. At 12 months, the BCVA was 57 letters, subretinal fluid had disappeared (g), and the retinal profile was normal except for minimally elevated retinal pigment epithelium (h).

Eyes were retreated when any of the following conditions occurred: (1) a visual acuity loss ≥5 ETDRS letters or unconsciously decreased vision; (2) the presence of subretinal fluid on OCT examination; (3) new bleeding macular lesions; and (4) increases in CNV lesion leakage or new lesions on FFA examination (Figure 2).

Data are presented as the mean ± standard error. All statistical calculations were performed using standard software (SPSS version 15.0, SPSS, Inc., Chicago, IL, USA). Visual acuity and CMT changes before and after treatment were compared using *t*-test. A chi-squared test was used to compare values between the groups. A *P* value of <0.05 was considered statistically significant.

## 3. Results

Baseline clinical characteristics of the two groups are summarized and compared in Table 1. No significant differences between the groups were observed, except in terms of BCVA and CMT (*P* < 0.05).

The changes in BCVA and CMT were monitored over time to determine and compare the efficacy of treatment in the two groups. Significant differences existed in BCVA and CMT between the groups at all time points (Table 2). This means that early treatment may lead to better visual improvement and greater reduction of macular leakage. At the last follow-up visit (12 months), 19 eyes (79.1%) in the early group and 10 eyes (50.0%) in the mid group had a visual gain of >15 ETDRS letters, and the difference was statistically significant (*χ* ^2^ = 4.130, *P* = 0.042).

IOP at baseline was normal in both groups at 13.9 ± 4.1 mmHg in the early group and 15.2 ± 3.9 mmHg in the mid group (*P* = 0.291). No significant differences were observed from baseline IOP values in either group at any time point examined. Additionally, there was no difference in IOP between the two groups at 1, 3, 6, 9, or 12 months. Although there were no significant changes in mean IOP from baseline, three eyes had an IOP of <10 mmHg on the first day after the injection (6 and 7 mmHg), and one eye had an IOP >21 mmHg (25 mmHg). All values normalized 3 days after injection without any drug intervention.

The average number of injections administered in the mid group was 2.53 ± 1.76 injections. This was significantly higher than the 1.22 ± 1.01 injections administered to the early group (*t* = 3.090,   *P* = 0.0035). No significant cataract formation was observed, and no serious complications from the injection procedure (e.g., endophthalmitis or retinal detachment) occurred.

## 4. Discussion

ICNV is not uncommon in Chinese patients, and it is one of the causes impairing visual function of young adults. It can sometimes lead to irreversible visual impairment. ICNV is highly prevalent in young women and usually affects only one eye [6]. It is conventionally treated mainly with PDT; however, PDT may cause temporary ischemia, can damage the retinal pigment epithelium (RPE) in the irradiated portion, can increase VEGF reactivity, and cause CNV lesions to recur [7, 8].

In recent years, ranibizumab became a first-line therapy for AMD [9]. Because ICNV and AMD have similar pathologies, ophthalmologists began using ranibizumab to treat ICNV after it was approved in China in 2012. While some patients responded well to this drug, others responded poorly, and relapse occurred in some others. We retrospectively evaluated our treated patients after dividing them into early and mid-groups according to disease duration and investigated the necessity and clinical efficacy of early treatment.

BCVA was significantly improved in both the early and mid-groups. Additionally, BCVA was statistically different between the two groups at all time points examined. CMT as measured by OCT was significantly reduced from baseline values in both groups at all follow-ups. There was a significant difference in CMT between the groups at all time points. At the last follow-up visit (12 months), 19 eyes (79.1%) in the early group and 10 eyes (50.0%) in the mid group had a visual gain of >15 ETDRS letters, and the difference was statistically significant (*χ* ^2^ = 4.130,   *P* = 0.042). Therefore, these data show comparable efficacy in the two groups. Over time, CNV progresses from bleeding and oozing to the gradual processes of scarring and fibrosis; therefore, early lesions have more reversible components; thus the treatment effect was superior.

The mean number of injections in the early group (1.22 ± 1.01) was significantly lower than that in the mid group (2.53 ± 1.76 injections, *P* = 0.0035). Therefore, it is possible that early injection of ranibizumab may reduce the number of injections required for ICNV treatment. Fewer injections mean lower costs, and this is particularly beneficial for patients who struggle to pay for the high cost of repeated injections.

IOP was also examined in this study. No significant differences were observed between pre- and posttreatment values. IOP was measured in both groups at all follow-up visits. Additionally, average IOP was not statistically different between the two groups at any time point examined. No serious side effects were noted in either study group.

The duration of ICNV mainly depends on the patients' attitude, which to some extent is subjective; however, patients employed in the present study were young adults, with typically good vision who were sensitive to their vision changes. Once decreased vision or visual distortion occurred, they could generally notice it in a timely manner. In addition, patients with other diseases causing visual impairment were excluded; this also helps to improve the accuracy of the estimated disease duration. Nevertheless, our study is limited by its small sample size and relatively short follow-up period.

## 5. Conclusions

This study shows that early treatment of ICNV can result in better visual prognosis, more obvious CMT decrease, and fewer numbers of injections over a 1-year follow-up period. Further studies with a longer follow-up period and a larger number of patients are warranted to further assess the efficacy and necessity of intravitreal injections of ranibizumab for ICNV.

## Figures and Tables

**Table 1:** A comparison of baseline clinical characteristics.

	Early group (*n* = 24)	Mid group (*n* = 20)	*P* value
Age (years)	31.8 ± 7.8	34.3 ± 8.9	0.326
Sex (male/female)	8/16	7/13	0.908
Subfoveal/juxtafoveal	13/11	12/8	0.697
Intraocular pressure (mmHg)	13.9 ± 4.1	15.2 ± 3.9	0.291
Refractive error (diopters)	−1.73 ± 1.9	−2.43 ± 2.1	0.253
Axial length (mm)	24.4 ± 1.4	24.8 ± 1.8	0.412
CMT before injection	368.2 ± 96.9	443.3 ± 126.1	0.031
BCVA (ETDRS)	67.9 ± 13.1	42.3 ± 24.8	0.001
Duration of CNV (days)	35.3 ± 16.9	114.8 ± 28.6	0.001

**Table 2:** Changes in best-corrected visual acuity (ETDRS), central macular thickness, and intraocular pressure during the 12-month monitoring period.

Time (months)	Change in ETDRS best-corrected visual acuity (letters)			Change in central macular thickness (*μ*m)			IOP (mmHg)		
	Early	Mid	*P*	Early	Mid	*P*	Early	Mid	*P*
1	21.8 ± 8.4	14.9 ± 9.7	0.015	185.3 ± 37.7	132.2 ± 32.3	0.0001	13.6 ± 4.8	13.4 ± 4.2	0.885
3	29.3 ± 9.9	18.4 ± 11.5	0.0016	182.4 ± 39.4	153.7 ± 31.2	0.011	13.5 ± 3.4	14.2 ± 2.5	0.449
6	35.2 ± 11.8	21.1 ± 13.1	0.005	196.4 ± 43.9	157.7 ± 49.7	0.0089	14.6 ± 2.6	13.9 ± 2.8	0.395
9	37.6 ± 15.3	25.8 ± 18.1	0.029	185.7 ± 46.8	117.3 ± 54.1	0.0001	13.4 ± 2.5	14.3 ± 3.2	0.301
12	36.9 ± 12.2	24.3 ± 16.2	0.005	195.3 ± 58.3	128.8 ± 43.3	0.0001	14.4 ± 3.1	15.3 ± 3.5	0.371

Data are presented as the mean ± standard error.

Changes in BCVA and CMT are compared to preinjection values.

IOP: intraocular pressure.
